# Deciphering the Association: Critical HDL-C Levels and Their Impact on the Glycation Gap in People Living with HIV

**DOI:** 10.3390/ijms26030914

**Published:** 2025-01-22

**Authors:** Elsa J. Anaya-Ambriz, Monserrat Alvarez-Zavala, Luz A. González-Hernández, Jaime F. Andrade-Villanueva, Sergio Zuñiga-Quiñones, Adriana Valle-Rodríguez, Tania E. Holguin-Aguirre, Karina Sánchez-Reyes

**Affiliations:** 1 Programa de Doctorado en Microbiología Médica, Centro Universitario de Ciencias de la Salud, Universidad de Guadalajara, Guadalajara 44340, Mexico; elsa.anaya@alumnos.udg.mx; 2 Departamento de Clínicas Médicas, Instituto de Investigación en Inmunodeficiencias y VIH, Centro Universitario de Ciencias de la Salud, Universidad de Guadalajara, Guadalajara 44350, Mexico; montserrat.zavala@academicos.udg.mx (M.A.-Z.); luceroga08@gmail.com (L.A.G.-H.); drjandradev@gmail.com (J.F.A.-V.); 3 Unidad de VIH, Hospital Civil de Guadalajara “Fray Antonio Alcalde”, Guadalajara 44350, Mexico; infectologosergio@gmail.com (S.Z.-Q.); adrianav.md@gmail.com (A.V.-R.); taniaholguinaguirre@gmail.com (T.E.H.-A.)

**Keywords:** HIV, T2D, HbA1c, fructosamine, G-gap, dyslipidemia

## Abstract

People Living with HIV (PLWHIV) present an increased risk of developing non-communicable diseases, such as type 2 diabetes (T2D), making it crucial to optimize glycemic control and assess metabolic markers. HbA1c is considered the gold standard for evaluating glycemic control, while fructosamine (FA) offers advantages in assessing non-glycemic determinants. Discrepancies between HbA1c and FA are common and may be influenced by temporal factors. The Glycation Gap (G-gap) emerges as a tool to clarify these discrepancies. A cross-sectional analytical study was conducted involving PLWHIV with various glycemic statuses, as well as patients with T2D and controls. Sociodemographic data were collected along with blood samples to measure biochemical profiles and FA. HbA1c predicted from FA (pHbA1c) was calculated using a linear regression equation, facilitating G-gap determination. A positive correlation was found between G-gap and levels of VLDL-C and triglycerides (TG). Additionally, a negative correlation was observed between HDL-C levels < 40 mg/dL and a positive G-gap. These associations suggest that the G-gap may be a useful tool for metabolic evaluation in PLWHIV and a preventive method for identifying individuals at risk of developing chronic complications related to T2D.

## 1. Introduction

People living with HIV (PLWHIV) have an increased risk of developing non-communicable diseases such as type 2 diabetes (T2D) due to the presence of HIV itself, the antiretroviral therapy (ART) and its side effects, the low-grade chronic inflammation triggered by HIV infection, among other factors. These factors, along with established lifestyle behaviors such as diet, physical activity, smoking, and aging contribute to the increased risk of developing cardiovascular disease (CVD) and their complications [1].

According to the Multicenter AIDS Cohort Study, the incidence of T2D in PLWHIV is fourfold greater than non-HIV controls [2]. Impaired glucose metabolism has been associated with altered levels of adipokines and alterations in CD4^+^ and CD8^+^ T-cell functions [3]. ART, mainly non-nucleoside reverse transcriptase inhibitors (NNRTIs), nucleoside reverse transcriptase inhibitors (NRTIs), and protease inhibitors (PIs), have been associated with metabolic alterations, including dyslipidemia and insulin resistance (IR) [4,5], while integrase strand transfer inhibitors (INSTIs) have been reported to promote weight gain. However, INSTI’s T2D risk is partially attenuated when adjusted for body mass index (BMI) changes and other variables [6]. Lastly, the altered inflammatory state is associated with posttranscriptional modifications in insulin signaling, IR, and a procoagulant state, which increases the risk of CVD [4].

T2D is a metabolic disorder strongly associated with atherogenic dyslipidemia, a lipid profile characterized by hypertriglyceridemia, reduced high-density lipoprotein cholesterol (HDL-C) levels, and an increased proportion of low-density lipoprotein cholesterol (LDL-C) particles [7]. This dyslipidemic pattern, prevalent in PLWHIV, elevates atherosclerosis and CVD risk [8]. In this population, alterations in lipoprotein metabolism are central to the increased cardiovascular risk observed in conjunction with T2D.

PLWHIV are characterized by a significant decrease in HDL-C, a well-established risk factor for CVD [9,10]. Recent studies analyzing the role of lipid metabolism and ART in glycemic control describe that ART implementation has a high level of dyslipidemia compared with HIV-negative subjects [11]. This effect on lipid metabolism has also been observed with the use of INSTIs [12]. Although they are currently the first-line therapy, INSTIs have been associated with an increase in glycated hemoglobin (HbA1c) levels [13] and a 31% increase in diabetes incidence when compared to other ART. These findings have been attributed to the weight gain observed after the use of INSTIs [14].

Regarding glycemic control, a retrospective study reported in PLWHIV on different ART regimens found a tendency of ART to increase fasting plasma glucose (FPG) and lipid profile levels, with three different patterns [15]. A meta-analysis reported a significant increase in FPG levels after 18 months of ART [16]. In addition, ART has been reported to cause IR [17].

Several mechanisms have recently been reviewed in relation to the effect of HIV and ART on lipid metabolism and glycemic control. Both ART-treated and ART-naïve PLWHIV have a significant decrease in genes related to cholesterol uptake, like CD36, HMGCR, SREBP2, among others, culminating in lower levels of HDL-C [18]. In addition, HIV’s Nef protein induces the degradation of the ABCA1, causing an accumulation of cholesterol in macrophages [18]. Low levels of HDL-C have been associated with IR [19], and in turn, hyperglycemia reduces HDL-C levels and function [20].

The optimization of glycemic control and the evaluation of metabolic markers are essential in the management of PLWHIV [21]. Currently, HbA1c is considered one of the diagnostic tests for T2D with a value ≥ 6.5% (≥48 mmol/mol) and a range of 5.7% to 6.4% (39–47 mmol/mol) for a state of pre-diabetes [22]. However, HbA1c measurements could underestimate the glycemic status of PLWHIV due to multiple factors. Some of these factors are the use of NNRTIs, NRTIs, and IPs, ART-related subclinical hemolysis, use of other medications like dapsone and ribavirin, hepatitis C virus (HCV) viremia, a low CD4^+^ T cell count (<500 cells/mm^3^), or the alterations in plasma proteins, including albumin [23,24,25,26,27,28].

Fructosamine (FA) is a ketoamine made from the covalent bond between glucose and the amino groups of plasma proteins, mainly albumin, and reflects the mean glucose concentration of the last 2–3 weeks prior to the measurement [29]. FA has proven useful in situations in which HbA1c is unreliable, such as variations in erythrocyte turnover and the presence of hemoglobin variants. FA has been proposed as an excellent alternative for evaluating glycemic control in PLWHIV [24].

HbA1c is the gold standard for assessing glycemic control due to its relationship with protein glycation, which is linked to the development of T2D complications [30]. However, changes in HbA1c cannot be explained only by glucose levels. This suggests the presence of non-glycemic factors that may influence HbA1c levels [31]. For the evaluation of the non-glycemic determinants of HbA1c, FA offers multiple advantages, including its great stability, the evaluation of the early response to the initiation of or change in treatment in T2D, as well as being a reliable measurement for patients with rapid changes in glucose homeostasis and larger glycemic excursions [32,33].

The normal range for FA is between 205 and 285 µmol/L [34], and changes indicate poor glycemic control in the weeks prior to the analysis. Improvements in FA tests have enhanced its specificity and demonstrated robust correlations with HbA1C levels. This has increased interest in FA as a complementary marker for T2D diagnosis, particularly in PLWHIV and other groups in which Hb1Ac may not be accurate [35].

Considering the above, several studies have demonstrated the need to consider alternative measurements of glycemic control to improve a timely diagnosis of preT2D or T2D and to carry out appropriate clinical interventions. HIV infection has been associated with a lower hemoglobin glycation index (HGI) [36]. Thus, HbA1c levels underestimate glycemic levels by 10% to 19% [37,38]. Based on the inaccuracy of HbA1c measurements in PLWHIV, FA determination has been proposed as a more accurate alternative for measuring average glycemic levels [27]. Furthermore, HbA1c has been correlated with FA = 0.72. When discrepancies were found, FA was elevated while HbA1c was at normal values (<6.5) [24,39].

On the other hand, discordance between HbA1c and other metrics for glycemic control, like FA, is common and may occur due to the equilibrium period of HbA1c or temporal factors [40]. In the context of HIV, temporal factors that may cause these discrepancies include fluctuations in the immune system due to HIV infection progression, response to ART, and changes in the patient’s overall health status over time. Additionally, the interaction between HIV and ART drugs may affect the patient’s metabolic physiology, potentially influencing the results of glycemic control tests such as HbA1c [38].

The calculation of the Glycation Gap (G-gap) emerges as a valuable tool to elucidate the observed discrepancies between HbA1c and FA levels. Cohen et al. conceptualized the G-gap as the disparity between observed HbA1c and the predicted HbA1c (pHbA1c) based on a regression analysis of FA levels. Under this definition, the G-gap is negative when the measured HbA1c is lower than the pHbA1c, and the G-gap is positive when HbA1c is higher [40]. In other words, the G-gap is proposed as a measure of the deviation of glycated HbA1c from its expected value, thus a positive G-gap means a higher than expected level of glycation and a negative G-gap indicates a lower level of glycation.

A positive G-gap is directly associated with diabetes complications like retinopathy, micro- and macro-vascular diseases, and mortality [41]. Hyperglycemia, inflammation, and oxidative stress are conditions that are elevated in PLWHIV and accelerate the process of glycation. Additionally, these conditions could directly impact the G-gap [30,42,43].

T2D is a significant comorbidity in PLWHIV and is considered the main cause of CVD, blindness, end-stage renal disease, amputations, and hospitalizations [44].

The G-gap’s clinical relevance lies in its capacity to monitor glycemic control when HbA1c levels are unreliable, a common case in PLWHIV. In addition, the G-gap itself has been associated with complications present in HIV infection or T2D, including renal, micro- and macro-vascular complications [41,45], leading to a reduced life expectancy for these populations. Importantly, there are no previous published reports of any relationship between the G-gap and complications in PLWHIV.

In this study, we aimed to investigate the association between the decrease in HDL-C and the G-gap in PLWHIV under different glycemic conditions. Elucidating the underlying pathophysiological mechanisms for the increased cardiovascular risk in PLWHIV may guide future clinical interventions.

## 2. Results

### 2.1. Sociodemographic and Anthropometric Profile

Eighty-two patients were included, distributed across the following groups: PLWHIV (n = 15), PLWHIV and pre-diabetes (PLWHIV + pre-T2D, n = 17), PLWHIV and T2D (PLWHIV + T2D, n = 17), people living with type 2 diabetes (PLWT2D, n = 18), and individuals without HIV or T2D as controls (n = 15). Of the included patients, 73.2% were male. The highest median age was observed in the PLWT2D group (57.5 years), ranging from 30 to 68 years. In contrast, the PLWHIV group had the lowest median age (37 years), with a range of 22 to 63 years (Table 1).

The mean weight was higher in the PLWHIV + T2D group (85 kg ± 17). The BMI was significantly increased in the PLWHIV + T2D and PLWT2D groups, with medians of 28.3 kg/m^2^ and 29.4 kg/m^2^, respectively. In total, 39% of the sample were overweight according to the BMI classification, while 36.6% were in the normal weight range. The waist-to-hip ratio (WHR) was obtained, and a significant increase was observed in the PLWHIV + T2D group, with significant differences noted when compared to the PLWHIV, PLWHIV + preT2D, and control groups (Table 1). The rest of the sociodemographic and anthropometric characteristics of the included patients are summarized in Table 1.

### 2.2. Clinical and Treatment Characteristics of PLWHIV

The median HIV viral load was 31, 20, and 38 copies/mL for PLWHIV, PLWHIV+pre-T2D, and PLWHIV + T2D, respectively. All groups had an absolute CD4^+^ T cell count median > 500 cells/µL and a nadir CD4^+^ T cell count median > 200 cells/µL (Table 2). Regarding ART, Biktarvy^®^ was the most common and stable therapy, used by 47 of the PLWHIV (95.9%) for an average period of 4.5 years (54 months) (Table 2). PLWHIV + preT2D was the group with the most ART switches, with the most common change being from NNRTI + NRTI co-formulation to INSTI. Regarding metformin treatment, the presence or absence of HIV infection did not alter the proportions of participants using metformin (82.4% and 83.3%, respectively) (Table 1).

### 2.3. Biochemical Characteristics

Evaluation of the glycemic profile revealed a significant elevation in glucose, HbA1c%, and FA levels in the groups diagnosed with T2D, both with and without HIV. In PLWHIV + T2D, the median glucose level was 139 mg/dL, with a range varying from 139 to 281 mg/dL. In PLWT2D, the median was 148 mg/dL, ranging from 79 to 262 mg/dL. Regarding HbA1c%, the PLWHIV+T2D group had a mean of 8.3%, with a standard deviation (SD) of 2.2, while the mean for the PLWT2D group was 7.8%, with an SD of 1.8. Additionally, the median FA level in the PLWHIV + T2D group was 404 µmol/L, with a range extending from 253 to 838 µmol/L. In contrast, the PLWT2D group had a median of 453 µmol/L ranging from 322 to 643 µmol/L. Finally, estimated average glucose (eAG) showed a significant increase in the groups diagnosed with T2D, regardless of HIV status.

Lipid profile was evaluated, but no significant differences between the groups were found, except for HDL-C, which showed a significant decrease in PLWHIV + T2D (median: 32 mg/dL; interquartile range: 26–45 mg/dL) compared to the rest of the groups. Though not significant, very low-density lipoprotein cholesterol (VLDL-C) and triglyceride (TG) values showed a tendency to increase in groups diagnosed with T2D, both with and without HIV (Table 3).

### 2.4. pHbA1c and G-Gap Calculations

**Figure 1:**
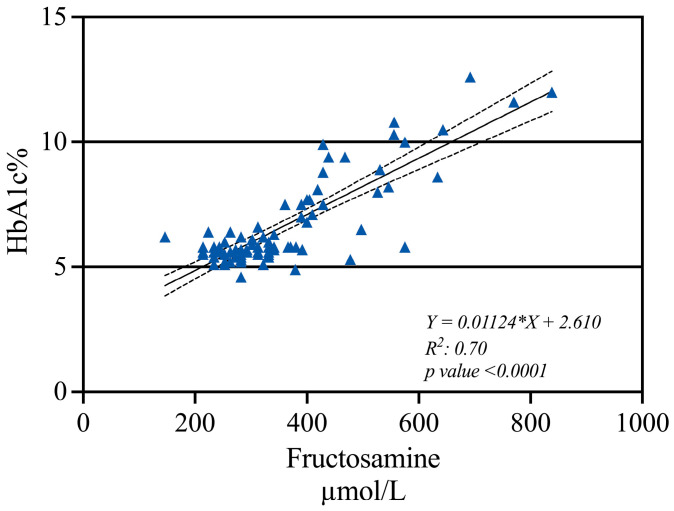
Linear regression of HbA1c% and FA using the whole study sample. The solid line indicates the linear regression, and the dotted lines represent 95% confidence intervals (Slope: 0.009663 to 0.01286; Y-intercept: 2.011 to 3.237; X-intercept: 333.2 to −157.3). The resulting equation was pHbA1c = 0.01124 * FA µmol/L + 2.610 (R2: 0.70).

In this study, the mean HbA1c% was 6.6%, with a 1.8 SD, and the median FA was 360 µmol/L, ranging from 146 µmol/L to 838 µmol/L. A significant correlation was observed between both variables, with a coefficient of determination (R^2^) of 0.7. To predict HbA1c values based on FA concentrations, linear regression analysis was performed with the whole sample, resulting in the following simplified equation: pHbA1c = 0.01124 * FA µmol/L + 2.610 (Figure 1).

**Figure 2:**
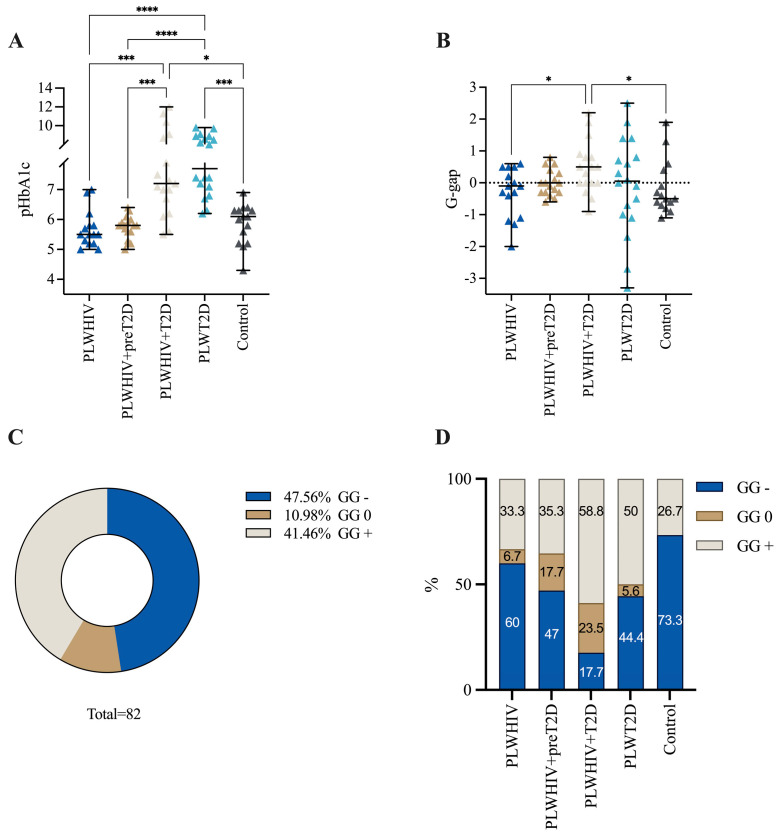
Calculation of pHbA1c and G-gap. (**A**) Scatter plots depicting (**A**) pHbA1C and (**B**) G-gap differences between groups. Results are shown as median and IQR. Cross-sectional comparisons were performed across the five study groups using a Kruskal–Wallis with a Dunn’s post hoc test. * *p* < 0.05, *** *p* < 0.001, and **** *p* < 0.0001. (**C**) Donut chart representing the distribution of G-gap values in this study; (**D**) stacked bar plot presenting the proportions of G-gap values by group.

pHbA1c was calculated using the equation derived from the linear regression between HbA1c% and FA. When comparing pHbA1c values among the groups, a significant increase was observed in the PLWHIV + T2D and PLWT2D groups when compared with all the other groups (Figure 2A). Subsequently, the G-gap was calculated, and a significant increase was observed in the PLWHIV + T2D group when compared to the PLWHIV group and control group (*p* = 0.0203 and 0.0103, respectively) (Figure 2B). In this study, 39 participants (47.56%) of the total sample showed a negative result in the G-gap, 9 (10.98%) had a G-gap equal to 0, and 34 (41.46%) showed a positive G-gap (Figure 2C). The distribution of the G-gap value in each group is illustrated in Figure 2D.

#### 2.4.1. Correlations Between G-Gap and Lipid Profile

**Figure 3:**
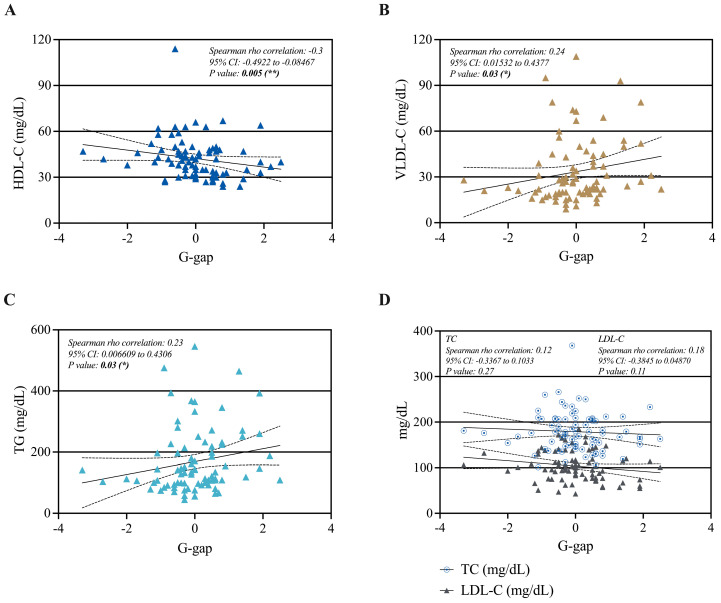
Spearman correlation between G-gap and each lipid profile variable, (**A**) HDL-C, (**B**) VLDL-C, (**C**) TG, (**D**) TC and LDL-C. A statistically significant correlation was defined by a *p*-value < 0.05 (* *p* = 0.03 and ** *p* = 0.005). TC: Total cholesterol, LDL-C: low-density lipoprotein cholesterol, HDL-C: high-density lipoprotein cholesterol, VLDL-C: very low-density lipoprotein cholesterol, TG: triglycerides, G-gap: Glycation Gap.

Using the whole sample, we looked for a correlation between the G-gap and the lipid variables. Spearman correlation analysis revealed a significant negative correlation between the G-gap and HDL-C (rho = −0.3; 95% CI: −0.4922 to −0.08467) (Figure 3A), a significant positive correlation for VLDL-C and TG (rho = 0.23; 95% CI: 0.01532 to 0.4377 and rho = 0.22; 95% CI: 0.006609 to 0.4306, respectively) (Figure 3B,C), and no significant correlation for total cholesterol (TC) or LDL-C (Figure 3D).

#### 2.4.2. Association of G-Gap with HDL-C Levels, HbA1c, and Combined Insulin Treatment

The sample was divided by their G-gap value: the G-gap- group (39 participants), the G-gap = 0 group (9 participants), and the G-gap+ group (34 participants). In total, 61.8% of the G-gap+ group and 33.3% of the G-gap- group had an HDL-C < 40 mg/dL. Regarding HbA1c, 73.9% of the G-gap+ and 40.7% of the G-gap- groups had an HbA1c > 7%. These differences demonstrated a significant association between HDL-C and HbA1C concentrations with G-gap (*p* = 0.010 and *p* < 0.001, respectively). Moreover, we identified more insulin usage in the G-gap+ group than in the G-gap- group (62.5% and 45.5%, respectively) (Table 4).

**Figure 4:**
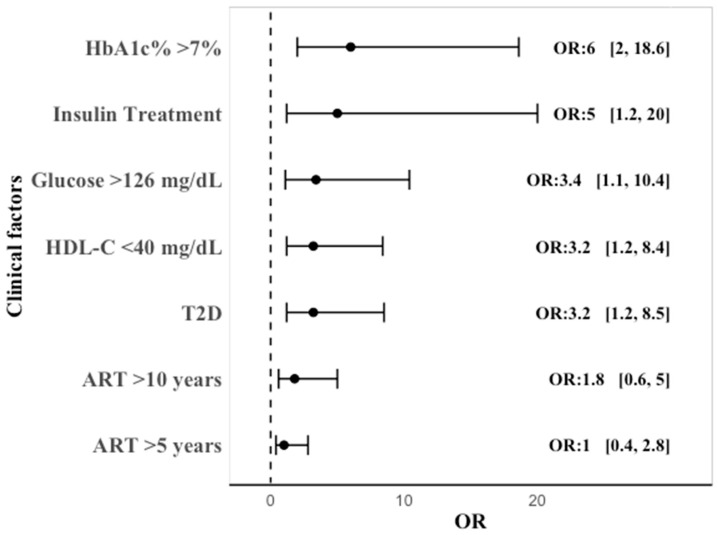
Association between several clinical factors and G-gap+. Forest plot illustrating the odds ratios (ORs) and 95% confidence intervals (brackets) for each clinical factor.

Lastly, we calculated the odds ratio (OR) between various clinical conditions and the presence of G-gap+. We identified a significant association with T2D (32, 95% CI: 1.2–8.5), insulin treatment (5, 95% CI: 1.2–20), plasma glucose > 126 mg/dL (3.4, 95% CI: 1.1–10.4), HbA1c > 7% (6, 95% CI: 2–18.6), and HDL-C < 40 mg/dL (3.2, 95% CI: 1.2–8.4). No significant association was found between G-gap+ and the duration of ART at two different time points over 5 or 10 years. These findings suggest that metabolic factors and insulin treatment are important predictors of G-gap+ (Figure 4).

## 3. Discussion

This is the first cross-sectional study to assess the discrepancies between HbA1c and FA in PLWHIV under different glycemic conditions, PLWT2D, and healthy controls. It was demonstrated that the G-gap has a significant negative correlation with HDL-C and a positive correlation with VLDL-C and TG. Additionally, G-gap+ was associated with HDL-C levels lower than 40 mg/dL, HbA1c% higher than 7%, and insulin usage. This suggests that discrepancies between HbA1c and FA may be related to the patient’s metabolic status, including the presence of dyslipidemia, which is considered a cardiovascular risk factor.

The increase in life expectancy among PLWHIV due to the use of ART has led to a series of consequences, including a significant rise in the prevalence of T2D. Currently, most deaths among PLWHIV occur due to complications from non-AIDS-related diseases such as CVD, one of the main complications of T2D [46]. Therefore, it has been suggested that PLWT2D should maintain adequate glycemic control and manage their lipid profile, which can be improved through lifestyle changes and pharmacological treatment with proper adherence [47]. According to previous reports, PLWHIV and T2D exhibited alterations in glucose levels and lipid profile, particularly a significant decrease in HDL-C [48]. Low levels of HDL-C have been shown to be a risk factor for CVD in the general population. In HIV infection, different ART regimens, including INSTIs, as well as the inflammatory state, can reduce HDL-C concentration and induce dysregulation in glucose metabolism and IR, all of them a precursor to T2D and increase the risk of CVD, independently of T2D [49,50].

HbA1c is a prominent marker for its ability to assess glycemic control. The American Diabetes Association (ADA) has recommended measuring this marker at least two times a year or every 3 months in individuals who do not meet therapeutic goals. This recommendation is because high levels of HbA1c (>7%) are related to long-term macro-vascular and micro-vascular complications, among others [51]. In this study, and regardless of HIV status, more than half of the PLWT2D did not achieve the recommended therapeutic goals and, therefore, are at higher risk of cardiovascular complications and mortality.

It has been reported that poor glycemic control is associated with factors such as gender, age, BMI, lipid profile, time of T2D diagnosis, blood pressure, T2D treatment, and the presence of comorbidities [52]. Interestingly, this study indicates that the type of treatment to lower TC and glucose, as well as BMI > 25 and dyslipidemia, are risk factors for poor glycemic control (HbA1c > 7%), particularly in PLWHIV. In accordance with our results, previous studies have reported a positive association between statin therapy and an increased risk of poor glycemic control through various mechanisms that have been discussed recently, among which are those that promote IR [53].

While HbA1c is the standard test for T2D diagnosis and monitoring, it has been shown to have certain limitations due to its failure to reflect individual variations between HbA1c and average blood glucose levels. It has even been reported to show discrepancies with the oral glucose tolerance test [54]. Additionally, the low-grade chronic hemolysis caused by HIV infection and ART (mainly NNRTI, NRTI, and PI) alters the glycemic status and the HbA1c values [25]. Other known factors that influence the measurement of HbA1c in PLWHIV are: a CD4^+^ T cell count < 500 cells/μL, drugs against opportunistic infections, like dapsone and ribavirin, and the presence of HCV [23,26,27,28].

In this study, only 15 patients had a CD4^+^ T cell count < 500 cells/μL and none of the patients had HCV or a recorded consumption of drugs that alter HbA1c, aside of ART. We had several PLWHIV with a regimen that included NNRTI, NRTI, or PI, mainly in the pre-T2D group. However, the scope of this study does not allow us to establish conclusions about the direct effect of ART on HbA1c, FA, or the G-gap. These findings suggest the existence of other factors that contribute to the discrepancies between HbA1c and FA in PLWHIV.

In cases in which HbA1c values may be altered, therapeutic decisions relying solely on HbA1c will most likely increase the risk of T2D and T2D-related complications. As mentioned earlier, FA emerges as an effective alternative for assessing average glucose levels over the past 2 or 3 weeks. Previous studies have documented a significant correlation between HbA1c and FA, even in PLWHIV individuals, and FA usage has become increasingly popular, although it is still not as widely used in PLWHIV [24].

Both PLWHIV + T2D and PLWT2D groups had FA levels above the cut-off of 293 µmol/L, which defines an inadequate glycemic control and predicts adverse outcomes [55]. Considering this cut-off point, 49 (59.8%) patients in the total cohort had high FA levels. Interestingly, 26 participants had high FA levels but HbA1c < 7%, indicating a discordant definition of inadequate glycemic control for our population. Of these 26 participants, 9 (34.6%) are in the control group, 6 (23.1%) in PLWT2D, 4 (15.4%) in PLWHIV + preT2D, 4 (15.4%) in PLWHIV + T2D, and 3 (11.5%) in PLWHIV. Thus, in PLWHIV, there could be an underestimation of inadequate glycemic control. Since the PLWHIV + preT2D group requires specific interventions to achieve optimal glycemic control, these results emphasize the importance of complementing HbA1c and FA values in T2D scrutiny.

A positive and significant correlation between FA and HbA1c was demonstrated in PLWHIV in different glycemic conditions in western Mexico. Interestingly, a correlation of 0.70 had already been reported in the USA population [24]. Additionally, a positive correlation has been previously reported in T2D with and without complications [40], as well as in other clinical conditions such as cancer [56].

The correlation between HbA1c and FA allowed the calculation of pHbA1c, which is necessary for calculating the G-gap. The G-gap is defined as the difference between the measured level of HbA1c and the pHbA1c [57].

This study demonstrates that 58.8% (10 cases) of PLWHIV + T2D individuals exhibited discordant results with G-gap+, indicating that the measured HbA1c levels were higher than those predicted by FA. In only 23.5% (4 cases) of PLWHIV + T2D individuals, a G-gap = 0 was observed, suggesting agreement between measured HbA1c and pHbA1c. On the other hand, 17.6% (three cases) of this group showed discordant G-gap- results, indicating that the measured HbA1c levels were lower than those predicted by FA. The G-gap is an important measure in diabetic patients since it has been demonstrated that a positive G-gap can predict diabetic complications such as nephropathy and retinopathy, which are considered the main complications of T2D [41].

Additionally, G-gap+ has been correlated with an elevated risk of CVD and mortality [41], and it has been considered a useful clinical marker for evaluating renal complications in T2D since the G-gap has been positively correlated with urinary albumin–creatinine ratio and macroproteinuria, and inversely correlated with the estimated glomerular filtration rate [58]. It has been reported that 40% of PLWT2D are expected to develop diabetic kidney disease [59]. PLWHIV has a prevalence of 3.5 to 48.5% for developing acute kidney injury and chronic kidney disease (CKD). We have previously reported a prevalence of 15.8% of CKD in our population [60]. Finally, PLWHIV have an increased risk of CVD. One of the best risk biomarkers for CVD is the WHR [61], which was significantly higher in PLWHIV + T2D, the same group with a high prevalence of patients with a G-gap+ and HDL-C < 40 mg/dL. These data suggest that the G-gap could be a useful tool for optimizing T2D management and ensuring accurate monitoring of glycemic control with the goal of reducing the risk of long-term complications [62].

Dyslipidemias are the most common risk factor for CVD, and they are frequently observed in patients with obesity, T2D, and HIV infection [63,64]. In this study, only the PLWHIV + T2D group had dyslipidemia based on a significant decrease in HDL-C < 40 mg/dL. Alterations in serum lipids in PLWHIV have been previously reported and associated with HIV infection, chronic use of ART, and the presence of inflammation [10]. In this context, a multicenter cohort study reported a decrease in TC, LDL-C, and HDL-C in PLWHIV from pre-seroconversion to the time before ART initiation. An increase in TC and LDL-C was reported 3 years after PI therapy [10].

The ART drugs most associated with lipid disorders are PI, NRTIs, and NNRTIs. INSTIs appear to have no direct impact on serum lipid levels, but few studies have been performed [65]. Currently, treatment guidelines for HIV infection recommend two NRTIs plus a third drug which may be an INSTI, NNRTI, or PI. In our study, 96% of PLWHIV were on ART with the drug Biktarvy^®^, and the presence of dyslipidemia is evident. However, this phenomenon cannot only be attributed to the current or previous ART regimen. In chronic HIV infection, it has been documented that the immune activation and inflammatory response promote an increase in lipid peroxidation and a decrease in plasma HDL-C by reducing ABCA1-dependent cholesterol efflux from macrophages, thereby interfering with the reverse transport of cholesterol from arteries and peripheral cells to the liver for excretion or recycling [18,66].

Additionally, glycation of HDL-C in PLWT2D is a factor related to the loss of the anti-inflammatory properties of HDL-C [66]. Research on the effect of lipoproteins and their association with CVD has described that PLWHIV often have low concentrations of HDL-C. This decrease correlates inversely with IL-6 and D-dimer and is also associated with a higher risk of CVD [65,66]. Thus, low HDL-C concentrations are associated with IR and chronic inflammation, which contribute to the risk of CVD.

This study shows a significant correlation of the G-gap with lipid profile parameters; in the case of HDL-C, a negative correlation was observed, rho = −0.29, while positive correlations were found with VLDL-C and TG. Additionally, we showed that among the 34 individuals with a positive G-gap, 61.8% had HDL-C concentrations < 40 mg/dL, 73.9% had HbA1c levels > 7%, 62.5% were receiving combined insulin therapy, and 36.4% were not undergoing insulin treatment.

Our results suggest a potential application of the G-gap in the clinic, as a positive G-gap provides a more objective measure of the glycation process in PLWHIV [41]. However, it is important to keep in mind that variability in protein turnover and obesity may affect the estimation of FA and the G-gap [41]. Currently, weight gain is common in these patients due to the use of INSTIs. On the other hand, the negative correlation of G-gap+ with low HDL-C levels and positive con VLDL-C and TG, highlights its potential as a marker to monitor dyslipidemia and other metabolic disorders in PLWHIV. In addition to our results, it has been described that glycation of LDL and HDL increases during hyperglycemia and inflammation, promoting atherosclerosis while hindering the anti-inflammatory and antioxidant effects of HDL-C [7,67]. The association of the G-gap with vascular complications and CVD has been evident, and its role as a good predictor has been emphasized in the literature [41,58].

As mentioned previously, the incidence of T2D in PLWHIV is up to four times higher than in the general population [2]. In addition, it has been described that HbA1c is perhaps not the best marker for glycemic monitoring of these patients [37]. Thus, it is necessary to find a good predictor of vascular complications for these patients. This is the first study to analyze the association between the G-gap and the dyslipidemia in PLWHIV. However, it is important to consider that this is a cross-sectional study, and several limitations should be addressed.

Firstly, with a sample size of 82 participants, the calculations of the linear regression of HbA1c% and FA, as well as each Spearman correlation for the G-gap and lipid profile, should be considered with care. Cohen et al., who made the first observation regarding the G-gap, calculated the HbA1c and FA regression with 153 patients [40]. However, studies reporting the relationship between the G-gap and T2D have been conducted with 104 patients [58], or even a smaller number of participants than the current study [68]. Regardless, our results serve as a foundation for future studies with a larger sample of PLWHIV to confirm our findings. Secondly, due to its cross-sectional design, we cannot confirm a causal relationship between HDL-C and the G-gap, and prospective studies are needed. Thirdly, the relationship between INSTIs and glucose metabolism, or T2D, remains unclear. Currently, INSTIs are considered a first-line therapy; hence, we believe longitudinal studies with different ART regimens with or without INSTIs are valuable in assessing the impact of all ARTs on the G-gap value and HDL-C. Fourthly, it has been mentioned that the G-gap can be a predictor of complications associated with intracellular glucose metabolism, as well as T2D-related complications and CVD [69]. As these associations cannot be identified in a cross-sectional study, prospective studies specifically aimed to confirm these issues are needed. Lastly, there is a possibility of residual confounding and selection bias to ART history prior to current therapy, as well as the overall length of ART treatment, mainly because of NNRTIs, NRTIs, and PIs on lipid and glycemic metabolism. In addition, it is important to consider HDL-C changes due to metformin, insulin, and statins, alone or in combination. Although further translational research and prospective clinical trials are needed before the G-gap may be incorporated into clinical practice, this study shows the promising potential of the G-gap to complement Hb1Ac for an adequate glycemic control of PLWHIV.

## 4. Materials and Methods

Design and Approval of the Study. This is a cross-sectional study conducted at the HIV and Immunodeficiencies Research Institute (InIVIH), University of Guadalajara, Guadalajara, Jalisco, Mexico. The study adhered to the Ethical Principles for Medical Research Involving Human Subjects outlined in the Helsinki Declaration of 1975 (as revised in Brazil 2013) and was approved by the Research Ethics Committee from the University Hospital of Guadalajara “Fray Antonio Alcalde” (HCG-FAA) with Approval No. HCG/CEI-0884/22 and Research Registration 138/22 as well as Research, Research Ethics and Biosafety Committees of the University Center of Health Sciences of the University of Guadalajara with Approval No. CUCS/CINV/0220/20; Research Registration 20-39. Written informed consent was obtained from each participant prior to enrollment.

Study population. Based on convenience sampling, a total of 82 adult participants were recruited from the HIV Unit from HCG-FAA, Guadalajara, Jalisco, Mexico. Additionally, PLWT2D and healthy controls were recruited. Inclusion criteria included: PLWHIV aged ≥ 18 and <65 years, on ART, with at least 1.5 years with an undetectable HIV viral load and with CD4^+^ T cells > 300 cells/μL, presenting different glycemic conditions, according to the criteria defined by the ADA [22]: normoglycemic (PLWHIV), pre-diabetic (PLWHIV + preT2D), or with T2D (PLWHIV + T2D). Exclusion criteria included: use of immunosuppressive drugs, corticosteroids, immunomodulators or antioxidants (30 days prior to recruitment), hepatotoxic drugs or use of herbs or herbal medicine (90 days prior to recruitment), use of thiazides, chronic use of acetylsalicylic acid, consumption of food supplements, minerals or vitamins (He, B12, C and E), use of atypical antipsychotics [22], chronic alcoholism, HBV+, HCV+, active SARS-CoV2 infection, dengue infection, chronic liver or kidney disease, splenectomy, hematological diseases or abnormalities (anemias or hemoglobinopathies), currently on hemodialysis, with recent blood loss or transfusion, patients in therapy with erythropoietin or with dehydrogenase-6-Phosphate deficiency. This information was validated in the electronic clinical record (SMART HIV database) of the HIV Unit. In the case of PLWT2D and healthy controls, glycemic condition was validated through the measurement of glucose and HbA1c at the Central Laboratory of Civil Hospital of Guadalajara “Fray Antonio Alcalde”, and exclusion criteria were checked with each participant and loaded in a database.

Sociodemographic and anthropometric characteristics. Sociodemographic and pathological data from PLWHIV were collected from the electronic clinical record (SMART HIV database) of the HIV Unit. A questionnaire was deployed to collect data from non-HIV participants. An anthropometric evaluation was performed (weight and height) to calculate their BMI using the formula BMI = Weight Kg/Height m^2^.

Biochemical profile. Blood samples were collected from all participants. After 30 min at room temperature, tubes were centrifuged at 1800× *g* for 10 min. The serum and plasma obtained were used immediately or aliquoted and stored at −80 °C until use. Glucose, HbA1c%, TC, LDL-C, HDL-C, VLDL-C, and TG were determined by photometry and colorimetric quantification (AU5800 autoanalyzer, Beckman Coulter^®^, Brea, CA, USA) in Central Laboratory of Civil Hospital of Guadalajara “Fray Antonio Alcalde”.

FA determination. FA concentrations were determined using a colorimetric assay with the FA Assay Kit (ab228558), (Abcam^®^, Cambridge, UK) following the manufacturer’s instructions. Absorbance (OD 530 nm) was measured using the spectrophotometer BioTek Synergy H1 (Agilent Technologies^®^, Santa Clara, CA, USA) values of the concentrations are expressed as µmol/L.

Mathematical measures. Mathematical equations were calculated using the following formulas:

- eAG=28.7×(HbA1c%)−46.7
- pHbA1c=0.01124×FA µmol/L+2.610, using the regression equation of HbA1c and FA.
- G−gap=Measured HbA1c%−pHbA1c [40].

Statistical analysis. A complete-case approach was conducted. Qualitative data are shown in frequency and percentage, and differences were analyzed using the Chi-Square Test w. For quantitative variables, normality was evaluated with the Shapiro–Wilk test. Mean and SD are shown for normally distributed variables. Otherwise, the median and interquartile range are shown. Comparisons between groups were conducted using ANOVA and Kruskal–Wallis tests, depending on the data distribution, followed by a post hoc analysis to identify significant differences.

For the calculation of pHbA1c, a univariate linear regression was performed between the percentage of HbA1c and FA levels. To identify correlations and associations, the Spearman correlation test and Fisher–Freeman–Halton exact test were utilized. A contingency table analysis (Crosstabs) was used to calculate the OR. A *p*-value < 0.05 was considered statistically significant. All statistical analyses were conducted using IBM SPSS Statistics^®^ for iOS [computer software], version 29.0.2.0, IL, USA, GraphPad Prism^®^ for iOS [computer software] version 10.1.1, CA, USA, under the license of Posit Software, PBC, and the R software^®^ [computer software] version 4.2.2.

## 5. Conclusions

This is the first study regarding the calculation of the G-gap in PLWHIV. The results demonstrated the association between G-gap+ and HDL-C levels < 40 mg/dL, HbA1c > 7%, and insulin treatment. These findings suggest that FA determination and G-gap calculation have the potential to be valuable tools for monitoring glycemic control in groups where HbA1C values are unreliable, as is the case in PLWHIV. Moreover, in PLWHIV, the G-gap could be used as a predictor of T2D-related complications such as CVD. However, due to the cross-sectional design, further studies are required prior to clinical application. We emphasize the need for longitudinal and prospective studies with a larger and more diverse population to validate G-gap as a reliable clinical marker for predicting cardiovascular risk in PLWHIV.

## Figures and Tables

**Table 1:** Sociodemographic and anthropometric characteristics of PLWHIV in different glycemic conditions, PLWT2D, and controls.

	PLWHIV	PLWHIV+ preT2D	PLWHIV+ T2D	PLWT2D	Control	*p*-Value
	(n = 15)	(n = 17)	(n = 17)	(n = 18)	(n = 15)	
Gender						
Women (%)	3 (20)	1 (5.9)	2 (11.8)	9 (50)	7 (46.7)	
Men (%)	12 (80)	16 (94.1)	15 (88.2)	9 (50)	8 (53.3)	0.007
Age (years)	37 (22–63)	50 (25–64)	50 (31–56)	57.5 (30–68)	39 (25–71)	0.009
Weight (Kg)	70 ± 9	74 ± 16	85 ± 17	78 ± 14	75 ± 16	0.065
Height (m)	1.7 (1.5–1.8)	1.7 (1.5–1.8)	1.7 (1.5–1.8)	1.7 (1.5–1.8)	1.7 (1.5–1.9)	0.807
BMI (Kg/m^2^)	24.5 (19–32)	24.6 (19–42)	28.3 (21–39)	29.4 (15–37)	26.5 (20–40)	0.022
BMI						
Underweight	NA	NA	NA	1 (5.6)	NA	
Healthy weight	8 (53.4)	9 (53)	4 (23.6)	2 (11.1)	7 (46.7)	
Overweight	5 (33.3)	3 (17.6)	7 (41.2)	10 (55.6)	7 (46.7)	
Obesity I	2 (13.3)	3 (17.6)	3 (17.6)	4 (22.1)	NA	
Obesity II	NA	1 (5.9)	3 (17.6)	1 (5.6)	NA	
Obesity III	NA	1 (5.9)	NA	NA	1 (6.6)	0.186
	0.87	0.86	0.97	0.93	0.85	
WHR	(0.6–1)	(0.8–1.3)	(0.85–1)	(0.77–1)	(0.75–0.98)	0.0027
T2D treatment						
Metformin (%)	NA	2 (11.8)	14 (82.4)	15 (83.3)	NA	<0.001
Insulin (%)	NA	NA	9 (52.9)	7 (38.9)	NA	<0.001
Statin	NA	NA	9 (52.9)	5 (27.8)	NA	<0.001

BMI: Body Mass Index, WHR: Waist-to-Hip ratio, T2D: type 2 diabetes. Qualitative data are presented as frequency and percentage; quantitative data with normal distribution are presented as mean ± standard deviation (SD); and quantitative data with non-normal distribution are presented as median and interquartile range (IQR). Analyses were conducted using ANOVA and Kruskal–Wallis tests, according to the distribution of the data. A *p*-value < 0.05 was considered statistically significant.

**Table 2:** Clinical and treatment characteristics of PLWHIV.

	PLWHIV	PLWHIV+ preT2D	PLWHIV+ T2D	*p*-Value
	(n = 15)	(n = 17)	(n = 17)	
Viral load	31	20	38	
(copies/mL)	(20–39)	(19–74)	(19–46)	0.45
Absolute CD4^+^ T cell (cells/μL)	541	592	583	
	(250–1310)	(301–2076)	(223–1151)	0.97
Nadir CD4^+^ T cell (cells/μL)	244	218	401	
	(15–675)	(11–1559)	(10–2181)	0.33
Current ART				
Biktarvy^®^ (%)	15 (100)	16 (94.1)	16 (94.1)	
Atripla^®^	-	1 (5.9)	-	-
Truvada^®^	-	-	1(5.9)	
Time-Biktarvy^®^	5	4.9	4.8	
(years)	(3.4–5.3)	(1.2–5.6)	(2.8–5.3)	0.66
History of ART prior to current.				
NNRTI + NRTI	5(55.6)	11 (68.8)	5 (41.7)	
PI + INSTI + NRTI	1 (11.1)	-	-	
INSTI + NRTI	1 (11.1)	-	2 (16.6)	-
NRTI + PI	2 (22.2)	5 (31.2)	5 (41.7)	
Time on ART prior to current (months)				
	30.7 ± 31.7	46.7 ± 35	34.8 ± 21.4	0.39
Total time on ART (years)	4.2	9.4	7	
	(1.2–19.8)	(0.8–19.8)	(1.7–23.3)	0.09

ART: antiretroviral therapy; NNRTI: Non-nucleoside reverse transcriptase inhibitor; NRTI: nucleoside reverse transcriptase inhibitor; PI: protease inhibitor; INSTI: integrase strand transfer inhibitor. Qualitative data are presented as frequency and percentage; quantitative data with normal distribution are presented as mean ± SD; and quantitative data with non-normal distribution are presented as median and IQR. Analyses were conducted using ANOVA and Kruskal–Wallis tests, according to the distribution of the data. A *p*-value < 0.05 was considered statistically significant.

**Table 3:** Biochemical characteristics of PLWHIV, PLWT2D, and healthy controls.

	PLWHIV	PLWHIV+ preT2D	PLWHIV+ T2D	PLWT2D	Control	*p*-Value
	(n = 15)	(n = 17)	(n = 17)	(n = 18)	(n = 15)	
Glycemic profile						
Glucose (mg/dL)	86 (69–101)	88 (72–103)	139 (80–281)	148 (79–262)	82 (70–99)	0.0001
HbA1c%	5.4 ± 0.4	5.7 ± 0.2	8.3 ± 2.2	7.8 ± 1.8	5.7 ± 0.3	0.0001
FA (µmol/L)	253	283	404	453	312	0.0001
	(214–392)	(214–341)	(253–838)	(322–643)	(146–380)	
eAG (mg/dL)	108 ± 11	118 ± 8	191 ± 63	178 ± 51	117 ± 9	0.0001
Lipid profile						
TC (mg/dL)	176	178	175	163	193	0.45
	(126–368)	(123–266)	(127–250)	(101–252)	(112–260)	
LDL-C (mg/dL)	106 ± 27	107 ± 28	101 ± 34	100 ± 37	105 ± 37	0.96
HDL-C (mg/dL)	42 (27–66)	43 (27–67)	32 (26–45)	43 (24–63)	48 (24–114)	0.0005
VLDL-C (mg/dL)	23 (9–74)	22 (11–67)	34 (15–109)	29 (15–60)	23 (14–95)	0.09
TG (mg/dL)	113 (45–368)	109 (56–333)	188 (76–546)	144 (67–302)	129 (71–476)	0.08

FA: Fructosamine, eAG: estimated average glucose, TC: total cholesterol, LDL-C: low-density lipoprotein cholesterol, HDL-C: high-density lipoprotein cholesterol, VLDL-C: very low-density lipoprotein cholesterol, TG: triglyceride. Reference values: Glucose: <125 mg/dL; HbA1c%: <6.5; FA: <285 µmol/L; TC: <200 mg/dL; LDL-C: <190 mg/dL; HDL-C: >35mg/dL; VLDL-C: 40 mg/dL; TG: <160 mg/dL. Quantitative data with normal distribution are presented as mean ± SD; and quantitative data with non-normal distribution are presented as median and IQR. Analyses were conducted using ANOVA and Kruskal–Wallis tests, according to the distribution of the data. A *p*-value < 0.05 was considered statistically significant.

**Table 4:** Fisher–Freeman–Halton exact test.

	G-Gap– (39)	G-Gap = 0 (9)	G-Gap+ (34)	*p*-Value
HDL-C < 40	13 (33.3)	7 (77.8)	21 (61.8)	
HDL-C > 40	26 (66.7)	2 (22.2)	13 (38.2)	**0.010**
HbA1c % ≥ 7	4 (17.4)	2 (8.7)	17 (73.9)	
HbA1c % ≤ 7	35 (59.3)	7 (11.9)	17 (28.8)	**<0.001**
Insulin treatment	3 (18.8)	3 (18.8)	10 (62.5)	
No insulin treatment	36 (54.5)	6 (9.1)	24 (36.4)	**0.035**

HDL-C: High-density lipoprotein cholesterol, HbA1c: glycated hemoglobin. The analysis was conducted using the Fisher–Freeman–Halton exact test. The values highlighted in bold indicate significant correlation with *p* < 0.05.

## Data Availability

The data presented in this study are available on request from the corresponding authors.
